# Knowledge and factors related to smoking among university students at Hodeidah University, Yemen

**DOI:** 10.18332/tid/109227

**Published:** 2019-05-16

**Authors:** Abdulsalam M. A. Nasser, Xinping Zhang

**Affiliations:** 1 School of Medicine and Health Management, Tongji Medical College, Huazhong University of Science and Technology, Wuhan, China

**Keywords:** related knowledge, associated, smoking prevalence, university students

## Abstract

**INTRODUCTION:**

Tobacco smoking, especially among university students, remains a significant issue worldwide. This survey aims to investigate and evaluate the smoking behavior and smoking-related knowledge and their relationship in students of Hodeidah University, Yemen.

**METHODS:**

A cross-sectional study was performed among students at Hodeidah University. Using a global youth tobacco survey and a global health professional survey, data were collected from three colleges (Commerce and Economics, Engineering, and Medicine) from April to June 2017, from 420 randomly chosen students.

**RESULTS:**

The smoking prevalence among university students was 33.1% (cigarettes 13.6%, waterpipe 9.3%, and 10.2% for dual cigarettes and waterpipe use), with a higher rate of smoking among males than females (36.3% vs 28.0%, p<0.001). The percentage of individuals participating in the three types of smoking among males and females, respectively, were 18.9% vs 5.0% for cigarettes, 1.9% vs 21.1% for waterpipe, and 15.4% vs 1.9% for dual cigarettes and waterpipe use, with a student mean age of 21.93 ± 2.55 years. The regression outcome revealed that year of study was highly associated with smoking (OR=0.87, 95% CI: 0.85–0.89, p<0.001). Age (OR=0.96, 95% CI: 0.94–0.99, p<0.05), residence (OR=1.05, 95% CI: 1.00–1.09, p<0.05) and family income (OR=1.03, 95% CI: 1.00–1.06, p<0.05) were also significant predictors of smoking.

**CONCLUSIONS:**

According to this study, most of the male students were cigarette users, while female students were waterpipe users. The prevalence of waterpipe use among females, as opposed to males, is an issue of concern. Policy makers may need to initiate anti-smoking programmes in Yemeni universities.

## INTRODUCTION

Tobacco use is among the most alarming problems concerning global health. Approximately 80% of smokers around the world are located within low-and middle-income countries where the highest rates of tobacco-related diseases and death are recorded^1^. Tobacco use is growing rapidly at a rate of about 3.4% per annum in the developing countries^2^. This is further evident in countries of Africa and the Eastern Mediterranean, where health systems are weak^3^.

Recent studies have reported that individuals who begin to smoke when they are teenagers and continue to smoke for another 10 to 15 years (as do 70%) could die twenty years earlier than expected^4^. The global tobacco-use prevalence among adults is estimated to be approximately 22%. Smoking rates in both middle- and upper-middle-income countries are similar, although rates are slightly higher in middleincome countries compared to upper-middle-income countries, 25% and 22%, respectively^5^.

Smoking prevalence increases in university students from their first to final years, raising concerns about the significance of sponsoring anti-smoking activities during the early years^6,7^. The Regional Report, Economics of Tobacco for the Middle East and North Africa Region (MNA), reported that the prevalence of youth smoking varied widely among some Arab countries from 7% in Oman to 53% in Lebanon, with 23% of adults being smokers, overall in the region^8^. The Palestinian Central Bureau of Statistics (PCBS) reported a high rate of smoking among youth (19.8%)^9^.

Smoking is common in Yemen among adult students. In a survey among high school and elementary school students, the Global Youth Tobacco Survey (GYTS) indicated that 6.8% were cigarette smokers (9.2% boys, and 2.5% girls), whereas 14.1% were waterpipe smokers with rates of 17.1% and 9.3% for boys and girls, respectively^10^.

The objective of this survey was therefore to investigate and evaluate smoking behavior and smoking-related knowledge among university students at Hodeidah University, Yemen.

## METHODS

### Study design and data collection

This cross-sectional study was conducted from April to June 2017, during the summer semester of the academic year. A questionnaire on smoking behavior and smoking-related knowledge was completed by students in three different academic departments, including one health-related college (Medicine) and two non-health-related colleges (Commerce and Economics, and Engineering) at Hodeidah University, Yemen.

The study population comprised full-time students of Hodeidah University. A total of 502 students of both genders enrolled in their first to last academic year participated in this study. The students were enrolled in Commerce and Economics, Medicine, and Engineering colleges and were randomly selected using stratified randomization. A total of 82 questionnaires were incorrectly filled and discarded, resulting in a total of 420 student participants who were included in this research for an overall response rate of 83.67%.

Four sections of 34 sets of questions were used to gather information about basic sociodemographic characteristics, smoking behavior among smokers, knowledge about some diseases caused by smoking and overall knowledge of health risks due to smoking (as detailed in the Results section). The questionnaire was prepared in Arabic and English to evaluate tobacco use, containing the global health professionals’ survey and the global youth tobacco survey^11,12^. Questionnaire forms were given to students to be filled in within 15 mins during a working day, after being informed that study participation was optional. The students were also informed not to display their names, and that all the collected data would be used for research purposes. The questionnaire comprised several variables including sociodemographic characteristics (age, sex, college, study levels, marital status, residence, monthly family income and smoking status) along with multiple yes/no questions and other questions with multiple levels.

This study was approved by the Research Ethics Committee of the Hodeidah University, Yemen, and Tongji Medical College, Huazhong University of Science and Technology, China.

### Definitions

Smokers were defined according to the criteria set forth by the WHO and Maziak et al.^13,14^ as those individuals who regularly smoked either one or more cigarettes per day, or one or more waterpipe per week, or less than one cigarette per day and likewise one or less waterpipe per week, at the time of the survey. The World Bank Country and Lending Groups (2018) consider Yemen as a lower-income economy according to the 2017 gross national income (GNI) per capita (≤US$995)^15^. The family monthly income (in US$) was then categorized into low (<100), average (100–300) and high (>300) groups.

### Statistical analysis

The IBM-SPSS statistics computer package version 24 was used for all analyses. Linear logistic regression was used for the data analysis, and chi-squared tests were performed to determine the significance and association between smoking and related factors. One-way ANOVA and pairwise t-tests were used to analyze smoking-related knowledge between colleges. All results were considered statistically significant at the 5% level of significance.

## RESULTS

### Characteristics

The study included 420 students, of which 259 (61.7%) were males and 161 (38.3%) were females; 60.0% were between 18 and 24 years old with a mean student age of 21.93 ± 2.55 years. Most students were in the College of Commerce and Economics (36.7%), followed by Engineering (32.1%) and Medicine (31.2%). Most participants were married (75.5%) and living with their parents (60.7%), and 56.9% of the students were from low-income families.

The smoking prevalence was higher (p<0.001) among students older than 24 years of age; the prevalence was 54.5% in students older than 24 years of age vs 44.8% in students less than 18 years old, and 21.0% in students aged 18–24 years. Senior students in their third and fourth years were more likely to smoke than junior students (p<0.001). When considering marital status, the highest smoking rate (50.5%) was among single students (p<0.001). A total of 139 students were classified as smokers; of these, 41.0% were cigarette smokers, 28.1% were waterpipe smokers, and 30.9% were dual cigarette and waterpipe smokers (p<0.001). The number of smokers was 139 (33.1%); out of the total population surveyed, 13.6% smoked cigarettes, 9.3% smoked waterpipes, and 10.2% smoked both cigarettes and waterpipe. A higher rate of smoking was recorded among males (p<0.001, 36.3% vs 28.0%). The percentage of cigarette smokers among males and females was 18.9% vs 5.0%, respectively. The percentage of waterpipe smokers was 1.9% vs 21.1%, respectively, among males and females, whereas for dual cigarette and waterpipe smoking, the percentage was 15.4% vs 1.9% for males and females, respectively (Table 1).

**Table 1:** Smoking status of the study sample based on the sociodemographic characteristics in university students, Yemen (N=420)

*Characteristics*	*Total*		*Smokers*	*Non-smokers*	*p*
	*n*	*(%)*	*n (%)*	*n (%)*	
**Sex**					
Male	259	61.7	94 (36.3)	165 (63.7)	0.088
Female	161	38.3	45 (28.0)	116 (72.0)	
**Age**					
<18	58	13.8	26 (44.8)	32 (55.2)	0.000
18–24	252	60.0	53 (21.0)	199 (79.0)	
>24 years	110	26.2	60 (54.5)	50 (45.5)	
**College**					
Commerce	154	36.7	55 (35.7)	99 (64.3)	0.160
Engineering	135	32.1	36 (26.7)	99 (73.3)	
Medicine	131	31.2	48 (36.6)	83 (63.4)	
**Marital status**					
Married	317	75.5	87 (27.4)	230 (72.6)	0.000
Single	103	24.5	52 (50.5)	51 (49.5)	
**Study level**					
First-year	203	48.3	67 (33.0)	136 (67.0)	0.000
Second-year	173	41.2	35 (20.2)	138 (79.8)	
Third-year	20	4.8	13 (65.0)	7 (35.0)	
Fourth-year	24	5.7	24 (100.0)	0 (0.0)	
**Residence**					
With family	255	60.7	79 (31.0)	176 (69.0)	0.252
Dormitory	165	39.3	60 (36.4)	105 (63.6)	
**Family income per month**					
Low	239	56.9	86 (36.0)	153 (64.0)	0.169
Average	143	34.0	45 (31.5)	98 (68.5)	
High	38	9.0	8 (21.1)	30 (78.9)	
**Smoking status**					
Cigarette	57	13.6	57 (100.0)	0 (0.0)	0.000
Waterpipe	39	9.3	39 (100.0)	0 (0.0)	
Cigarette + waterpipe	43	10.2	43 (100.0)	0 (0.0)	
Nothing	281	66.9	0 (0.0)	281 (100.0)	

The results from the logistic regression analysis (Tables 2 and 3) reveal that individuals of younger age (OR=0.96, 95% CI: 0.94–0.99, p<0.05) and who were in an earlier year of study (OR=0.87, 95% CI: 0.85–0.89, p<0.001) were less likely to be smokers. Individuals who resided in a dormitory (OR=1.05, 95% CI: 1.00–1.09, p<0.05) and who had a low family monthly income (OR=1.03, 95% CI: 1.00–1.06, p<0.05) were more likely to be smokers. However, sex, marital status and college had no significant association with smoking (p>0.05).

**Table 2:** Relationship between sociodemographic factors and smoking among university students, Yemen (N=420)

*Variable*	*SE*	*p*	*OR ( 95% CI)*
Sex	0.0210	0.153	1.03 (0.99–1.07)
Age	0.0144	0.007	0.96 (0.94–0.99)
Marital status	0.0214	0.262	0.98 (0.94–1.02)
Study level	0.0110	0.0001	0.87 (0.85–0.89)
Residence	0.0212	0.025	1.05 (1.00–1.09)
Family income per month	0.0131	0.035	1.03 (1.00–1.06)
Colleges	0.0108	0.279	1.01 (0.99–1.03)

SE: standard error, OR: odds ratio, CI: confidence interval.

**Table 3:** Smoking behavior among smokers and its relation to type of college (n=139)

*Item*	*Commerce and Economics*	*Engineering*	*Medicine*	*Total (%)*	*p*
	*n (%)*	*n (%)*	*n (%)*		
**First cigarette smokers by age**					
<12	5 (26.3)	5 (26.3)	9 (47.4)	19 (13.7)	0.037
12–18	23 (37.1)	12 (19.4)	27 (43.5)	62 (44.6)	
>18 years	27 (46.6)	19 (32.8)	12 (20.7)	58 (41.7)	
**How long have you been smoking?**					
<1	0 (0.00)	1 (12.5)	7 (87.5)	8 (5.8)	0.000
1–3	12 (24.0)	17 (34.0)	21 (42.0)	50 (36.0)	
3–5	24 (63.2)	9 (23.7)	5 (13.2)	38 (27.3)	
>5 years	19 (44.2)	9 (20.9)	15 (34. 9)	43 (30.9)	
**How many cigarettes do you smoke a day?**					
<5	11 (32.4)	7 (20.6)	16 (47.1)	34 (24.5)	0.004
5–10	6 (22.2)	6 (22.2)	15 (55.6)	27 (19.4)	
10–20	6 (35.3)	4 (23.5)	7 (41.2)	17 (12.2)	
>1 pack	32 (52.5)	19 (31. 1)	10 (16.4)	61 (43.9)	
**Do you think it is possible to quit smoking?**					
Yes	54 (46. 2)	24 (20.5)	39 (33.3)	117 (84.2)	0.001
No	1 (4.5)	12 (54.5)	9 (40.9)	22 (15.8)	
**Did you try to stop smoking?**					
Yes	20 (39.2)	10 (19.6)	21 (41.2)	51 (36.7)	0.209
No	35 (39.8)	26 (29.5)	27 (30.7)	88 (63.3)	
**Will you accept help to quit smoking?**					
Yes	43 (40.2)	30 (28.0)	34 (31.8)	107 (77.0)	0.233
No	12 (37.5)	6 (18.8)	14 (43.8)	32 (23.0)	
**Previous attempts to quit smoking**					
Never	38 (36.2)	30 (28.6)	37 (35.2)	105 (75.5)	0.317
1	0 (0.00)	1 (33.3)	2 (66.7)	3 (2.2)	
2	1 (50.0)	0 (0.0)	1 (50.0)	2 (1.4)	
3	5 (71.4)	1 (14.3)	1 (14.3)	7 (5.0)	
>3	11 (50.0)	4 (18.2)	7 (31.8)	22 (15.8)	
**Longest abstinence time from smoking**					
Within 1 week	29 (38.2)	16 (21.1)	31 (40.8)	76 (54.7)	0.001
Within 1 month	4 (15.4)	13 (50.0)	9 (34.6)	26 (18.7)	
Within 3 months	9 (42.9)	5 (23.8)	7 (33.3)	21 (15.1)	
Over 3 months	13 (81.3)	2 (12.5)	1 (6.3)	16 (11.5)	

As shown in Table 3, when stratifying the timing of the first cigarette smoked by age, the results were statistically significant (p<0.05). Most smokers started cigarette smoking between 12–18 years (44.6%) while for ages >18 years they were slightly less (41.7%). Higher percentages of Medicine students started smoking in the age range of <12 (47.4%) and 12–18 years (43.5%) compared to the other two colleges.

### Student knowledge about some ill effects and toxic chemicals from cigarettes

As shown in Table 4, there were significant differences among students of the different colleges with regard to level of knowledge about the effects of smoking (p<0.05) as well as on toxic tobacco substances (p<0.05). Overall, there was a significant difference between the three faculties in the total level of knowledge related to smoking (p<0.05).

**Table 4:** Knowledge of Yemeni university students about some ill effects caused by cigarette smoking and toxic chemicals from cigarettes

	*One-way ANOVA results*					*Pairwise comparison t-test (results between colleges)*			
*Colleges*	*n (%)*	*Mean*	*SD*	*F*	*p*	*College pairs*	*MD*	*95% CI*	*p*
**Knowledge about some ill effects**									
Economics	154 (36.7)	5.903	3.509	3.727	0.025	1 – 2	-0.846	(-1.836, 1.453)	0.123
Engineering	135 (32.1)	6.748	3.420			1 – 3	0.269	(-0.730, 1.268)	1
Medicine	131 (31.2)	5.634	3.559			2 – 3	1.115	(0.084, 2.145)	0.029
**Knowledge about some toxic chemicals**									
Economics	154 (36.7)	3.2208	2.23533	5.351	0.005	1 – 2	-0.764	(-1.837, -0.142)	0.01
Engineering	135 (32.1)	3.9852	2.08758			1 – 3	-0.031	(-0.659, 0.596)	1
Medicine	131 (31.2)	3.2519	2.25778			2 – 3	0.733	(0.086, 1.381)	0.02
**Total-related knowledge**									
Economics	154 (36.7)	9.1234	4.81871	5.554	0.004	1 – 2	-1.610	(-3.020, -2.004)	0.019
Engineering	135 (32.1)	10.7333	4.83951			1 – 3	0.238	(-1.183, 1.659)	1
Medicine	131 (31.2)	8.8855	5.28079			2 – 3	1.848	(0.382, 3.314)	0.008

1: Economics College, 2: Engineering College, 3: Medicine College. MD: mean difference, SD: standard deviation.

There was no statistically significant difference among the Commerce college and Engineering college as well as among the Commerce college and Medicine college in the level of knowledge about the impact of smoking. While the difference was significant between the Engineering college and Medicine college (p<0.05). Engineering college students had a higher level of knowledge.

There was a significant difference among the Commerce college and Engineering college (p<0.001), in the level of knowledge of tobacco toxic substances. Engineering students’ knowledge level was higher than that of the Commerce college students. Also, there was no significant difference between the Commerce college and Medicine college in the knowledge level of tobacco toxic substances. While the difference was statistically significant among the Commerce college and Medicine college in the knowledge level of tobacco toxic substances (p<0.05), the Engineering students’ knowledge level was higher than that of the Medicine students.

Overall, the Commerce college and Engineering college had statistical significance in the level of smoking-related knowledge (p<0.05) with Engineering students having better knowledge. Moreover, there was no significant difference between the Commerce college and Medicine college in knowledge, while the Engineering college and Medicine college had statistical significance difference in the level of smoking-related knowledge (p<0.05) (Table4).

## DISCUSSION

In the current study, the results show a higher prevalence rate of smoking among students aged 18- 24 years than shown by previous studies in some Arab countries, such as Libya (28.3%), Syria (11%)^16,17^ and non-Arab countries, such as Cameroon (11.2%) and India (20.2%)^18,19^. The differences between Yemeni students and the other populations may be due to differences in cultural and social habits that ascribe different meanings to being a smoker.

The results from this study show a higher prevalence of smoking with almost equal proportions among both genders compared to those from Saudi Arabia and Palestine, where a higher prevalence was observed among males than females (29.4% vs 11.4%) and (52.7% vs 16.5%), respectively^20,21^. This finding may be attributed to the lack of awareness or comprehension of smoking risks to health in addition to the sales promotion by tobacco commercials that exert increased pressure on young people due to civil war and poor economic situations in Yemen.

In this study, the majority of females, compared to males, were waterpipe smokers. The findings related to the prevalence of waterpipe smoking in this research are similar to those of previous studies among students in rural areas of Yemen in 2018^22^. These results differ from those found in a study conducted in Jordan^23^. The predominance of waterpipe smoking among females in this study reflects the replacement of cigarette smoking by waterpipe smoking as a sign of prestige among young people in the Eastern Mediterranean Region^24^; moreover, waterpipe smoking is considered more acceptable than cigarette smoking for women^25^.

This study also found that smoking prevalence increased with age, producing a significant association between student age and the tendency to smoke. It is obvious that most smokers were over 24 years old, as confirmed by similar studies^26,27^, which might be due to family pressure against youth smoking that lessens as individuals acquire more freedom.

This study indicates that the prevalence of smoking increases significantly with years of study; thus, senior students in their third and fourth years had a higher prevalence of smoking than did junior students. This may be due to the longer exposure of senior students to older smokers within the university environment (friends, teachers, employees, etc.) who could strongly influence their attitudes. This indicates that students in the final and second-to-final study years warrant specific training for smoking cessation.

In regard to marital status, smoking prevalence was obviously higher among single individuals than among married individuals; this suggests that there are greater psychological, economic and social pressures on students who live in dormitories to smoke, and single students may smoke for comfort or when influenced by friends. Similar findings were obtained by studies in Japan and Albania^28,29^.

In this study, the majority of smokers expressed a desire to quit smoking, and the largest percentage of students from all three colleges reported that the longest abstinence time from smoking was one week.

### Limitations and strengths

The study covered three different academic colleges, one health-related college (Medicine) and two non-health-related colleges (Commerce and Economics, and Engineering) at Hodeidah University. Hence, the results can be generalized to similar areas with similar social characteristics. However, this study is considered the first of its kind as a student-based survey on smoking prevalence and associated factors in Hodeidah University and Hodeidah Province. Since the participants were students of Hodeidah University, the findings of this study are not applicable for smoking prevalence prediction in other Yemeni universities. Furthermore, selection bias and information bias cannot be excluded.

## CONCLUSIONS

Smoking prevalence in students of Hodeidah University was relatively higher than previous rates reported for other Yemenis. There was a statistically significant association of smoking with age, year of study, residence, and family income. According to this study, the majority of males were cigarette smokers while the majority of females were waterpipe smokers. The predominance of waterpipe smoking among females is a concern. The results urge policy makers to initiate anti-smoking programs. University students in Yemen should receive health education and counselling to reduce smoking habits in higher education institutions.
